# Understanding of remora's “hitchhiking” behaviour from a hydrodynamic point of view

**DOI:** 10.1038/s41598-021-94342-x

**Published:** 2021-07-21

**Authors:** Yunxin Xu, Weichao Shi, Abel Arredondo-Galeana, Lei Mei, Yigit Kemal Demirel

**Affiliations:** 1 Department of Naval Architecture, Ocean & Marine Engineering, University of Strathclyde, Henry Dyer Building, 100 Montrose Street, Glasgow, G4 0LZ UK; 2 School of Ocean Engineering, Harbin Institute of Technology (Weihai), No. 2 Wenhua West Road, Weihai City, Shandong Province China

**Keywords:** Mechanical engineering, Biomechanics

## Abstract

Symbiotic relationships have developed through natural evolution. For example, that of the remora fish attached to the body of a shark. From the remora’s perspective, this could be associated to an increased hydrodynamic efficiency in swimming and this needs to be investigated. To understand the remora's swimming strategy in the attachment state, a systematic study has been conducted using the commercial Computational Fluid Dynamics (CFD) software, STAR-CCM + to analyse and compare the resistance characteristics of the remora in attached swimming conditions. Two fundamental questions are addressed: what is the effect of the developed boundary layer flow and the effect of the adverse pressure gradient on the remora’s hydrodynamic characteristics? According to the results, the resistance of the remora can generally be halved when attached. Besides, the results have also demonstrated that the drag reduction rate increases with the developed boundary layer thickness and can be estimated using the boundary layer thickness ratio and velocity deficit. The paper demonstrates that the most frequent attachment locations are also the areas that provide the maximum drag reduction rate.

## Introduction

**Figure 1:**
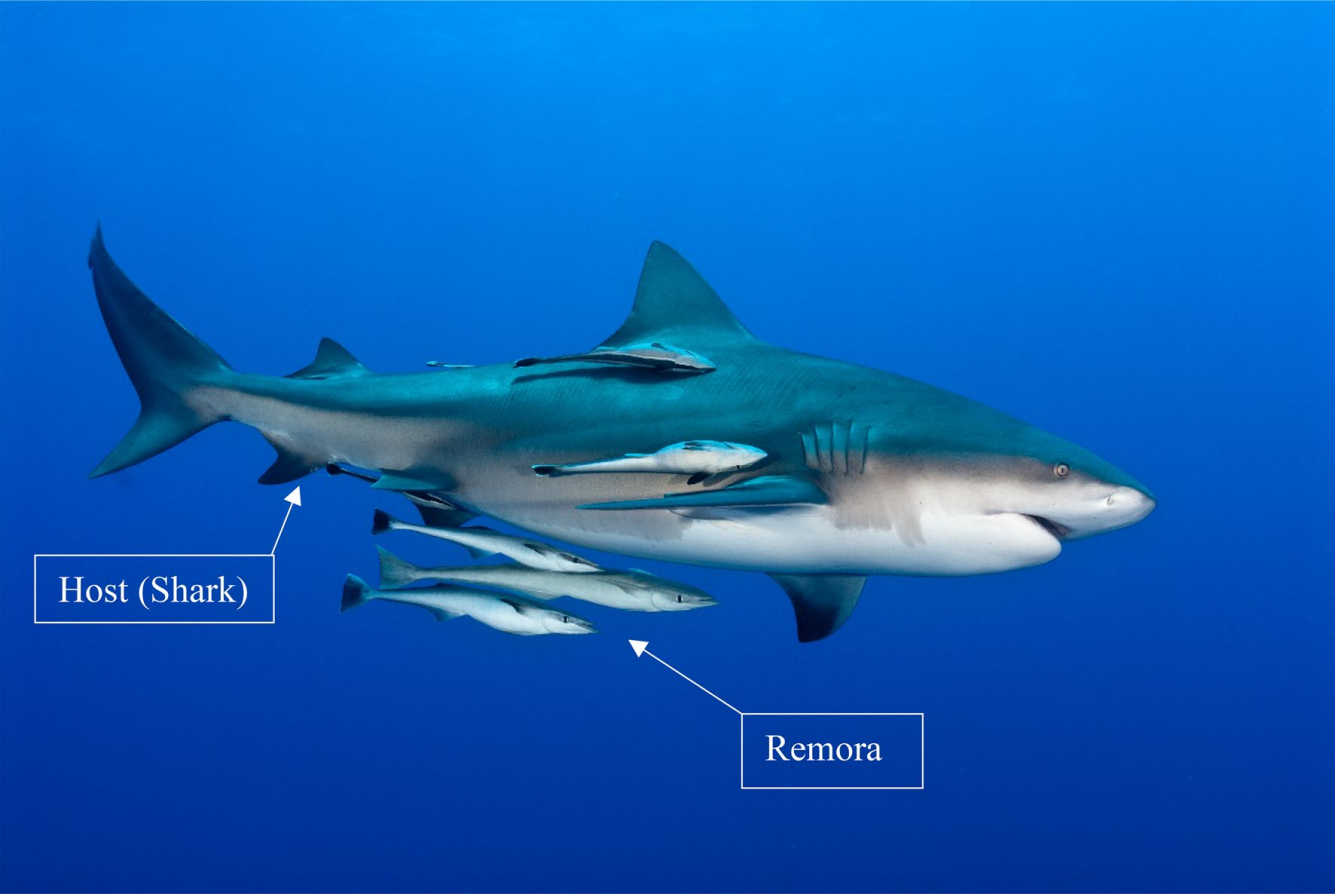
The attached swimming of remora with the host (created by Fiona Ayerst/Shutterstock.com).

Natural cooperative relationships have developed throughout the evolution process. Remora fish (e.g., *Echeneis neucratoides*, sometimes called “suckerfish”) are considered to be one of the laziest fish in the ocean, as they are more likely to be found attached to a host rather than swimming on their own. This is shown in Fig. 1^1–3^. In fact, the remora fish has a rather poor swimming ability. But because they “hitchhike”, they can travel over long distances. The hosts of the remora include a wide range of marine living creatures, such as sharks, turtles, as well as man-made marine vehicles.

**Figure 2:**
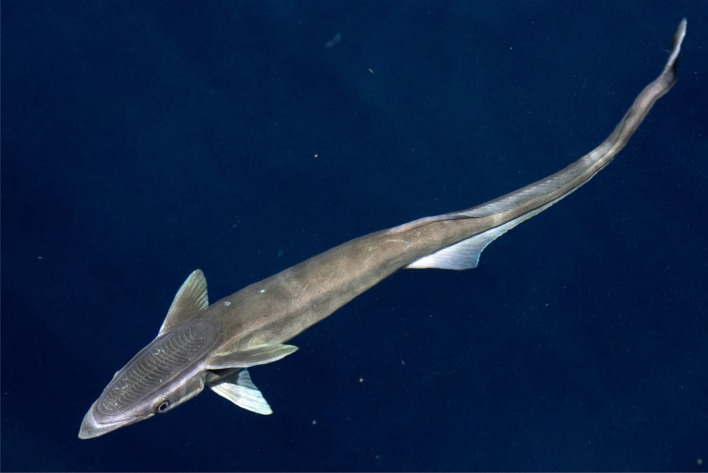
The suction disc of the remora (created by Andrea Izzotti/Shutterstock.com).

The remora’s ability to attach to a host is accredited to their suction disk, as shown in Fig. 2, a modified oval, sucker-like organ with slat-like structures to create suction, on which there are numerous parallel pectinated lamellae strengthening the adhesion to the surface of the host^3^. At the same time, the soft lip on the rim of the sucking disc of the remora is capable of performing a perfect mating onto the varying attachment positions to improve suction^4^. Dolphins usually leap out of the sea, spin in the air, and hit back the water surface to remove the remora fish by using both the centrifugal force and the water impact force^5^. However, this does not occur with sharks, where the remora remains mostly undisturbed.

In addition, the remoras are able to memorise their previous attachment locations through touch receptors in the sucking discs^6,7^. Furthermore, the attachment location is not selected at random. Brunnschweiler^8^ found that nearly 40% of the attachments (137 out of 345) have been performed near the sharks’ belly area, followed by 27% at the back and with the lowest number of attachments in the caudal fin area. This has attracted a number of researchers to study the reasons for such behaviour. From a marine biology point of view, it was suspected that the remora usually tends to select a position that does not irritate the host^9^. Meanwhile the remora tends to select locations with the minimum motion and deformation while the host is swimming in order to have a stable platform^1^. However, currently there is a lack of understanding about the hydrodynamic merits of selecting these attachment locations. Based on the study conducted by Brunnschweiler, 2006, it seems like the remora fish have learnt to seek for areas with adverse pressure gradients and developed boundary layers. Therefore, whether the remora fish selects its attachment location based on hydrodynamic criteria still needs to be investigated. By researching the hydrodynamic characteristics of the remora on varying attachment locations, the remora’s unique behaviours could be applied to autonomous underwater vehicles (AUVs), which currently cannot perform docking and recovery without asking the mother vehicle to come for a halt.

Under this framework, the study researched into the hydrodynamic mechanism of the remora fish swimming to understand its behaviour from a hydrodynamic point of view. Firstly, based on the literature a generalised remora fish model and a shark model as the host are established. Subsequently, the simulations in this study are divided into three cases, which are: (1) the free-swimming case, (2) the flat plate boundary layer case and (3) the host attachment case. Firstly, the free-swimming case mainly serves as a reference case to set the benchmark and understand the individual hydrodynamic performance of the remora and the shark. Secondly, the flat plate boundary layer case is set up to study the relationship between the drag on the remora and the independent variables, including the boundary layer thickness, the incoming flow velocity and the associated Reynolds number. This study is useful to understand the effect of boundary layer flow on the drag of remora fish. Thirdly, the attachment location case reveals the effect of the boundary layer flow in combination with the pressure gradient. By combining these three cases, a comprehensive understanding can be achieved for the remora’s hitchhiking behaviour.

## Model information and geometry preparation

The section below describes the details of the simulated models for the remora fish and the shark model. The models were created based on the natural shape and sizes of the actual remora and shark but adapted to be suitable for CFD simulation, and rigid models were used.

### The remora fish model

**Figure 3:**
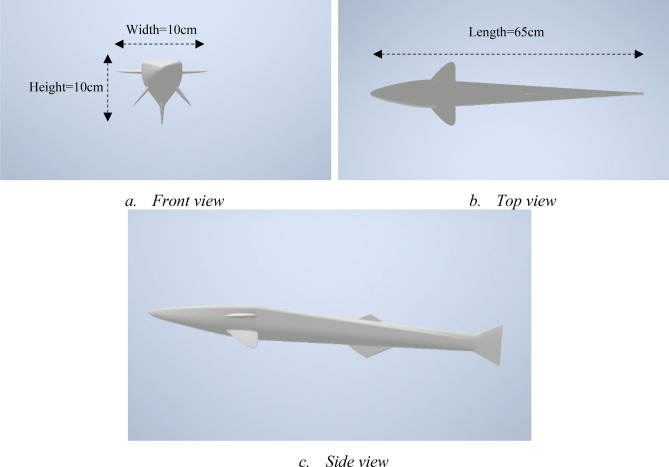
The 3D remora fish model for CFD simulation (Created using Autodesk Inventor 2020).

The remora fish model is created based on the natural form and principal parameters of the remora living in the Puerto Rico sea area. According to the literature, the length of the remora in the Puerto Rico sea area is between 51 to 73 cm, therefore in this study, the length of the remora model is selected to be an average size of 65 cm^10^. It is to note that when the remora attaches to the host, four pectoral fins of the remora open, with two of them staying close to the host. This will be modelled accordingly in the simulation. Furthermore, when the remora's suction disc attaches on the surface of an object, the body of the remora will be set at an angle, about 5°. Interestingly, we note that when the remoras swim on their own, their suction discs face upwards. In the simulation, the critical hydrodynamic features must be maintained (e.g., pectoral fins), while features posing minimal hydrodynamic effects can be simplified (e.g., scales). Based on the above principles, a 3D remora model has been built for the following CFD simulation. Basic parameters of the remora fish model are presented together with the 3D CAD model in Fig. 3.

### The shark model

**Figure 4:**
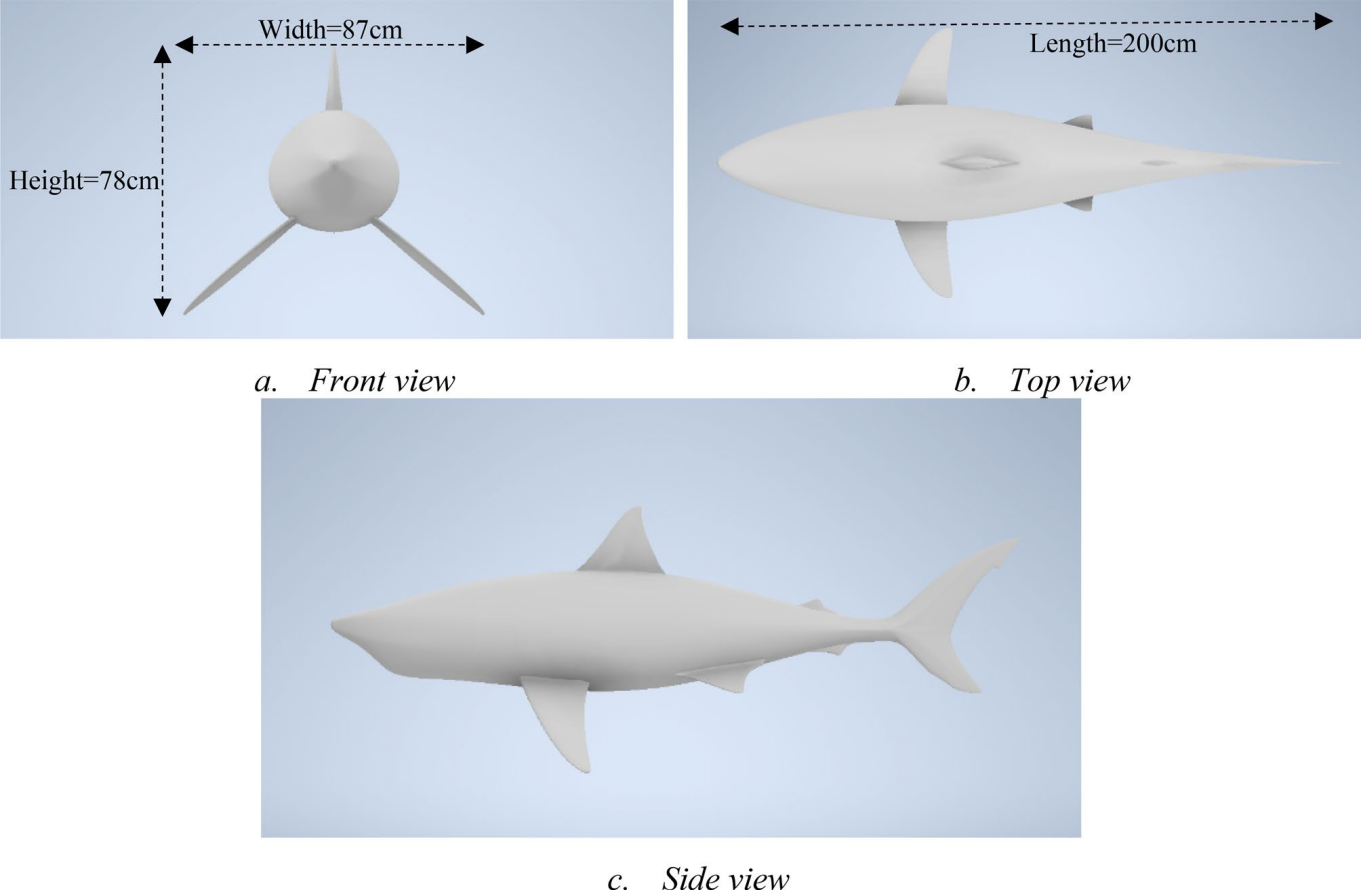
The 3D CAD oceanic whitetip shark model used for CFD simulation (Created using Autodesk Inventor 2020).

The oceanic whitetip shark, which swims also in the Puerto Rican area^11^, was selected with an average length of 2 m^12^. Similarly to the remora model, the original shark model is simplified for the CFD simulation. Figure 4 shows the basic parameters of the shark model.

## CFD method and free-swimming simulations

Numerical simulations were performed with the CFD software STAR-CCM + to investigate the hydrodynamic free-swim characteristics of the remora and the shark and to set out our benchmark cases. The average speed of the oceanic whitetip shark ranges between 1.2kn to 1.4kn with a maximum speed of 8.9kn^13^. Since the remora attaches onto the shark, the remora can experience the same speed as well. Therefore, in this research, the velocities of the remora are set to vary from 1 to 8kn. During the simulation, the drag characteristics are calculated, and the following methodology is adopted.

### CFD simulation setup

A Reynolds-averaged Navier–Stokes (RANS) model and a K-ω Shear Stress Transport (K-ω SST) turbulence model were chosen for this study^14^. For the wall treatment, we utilized the all y + setting in STAR-CCM +. This is a hybrid approach that implements a high-y + treatment (y +  > 30) for coarse meshes and low-y + treatment (y +  < 1) for fine meshes^15^.

**Figure 5:**
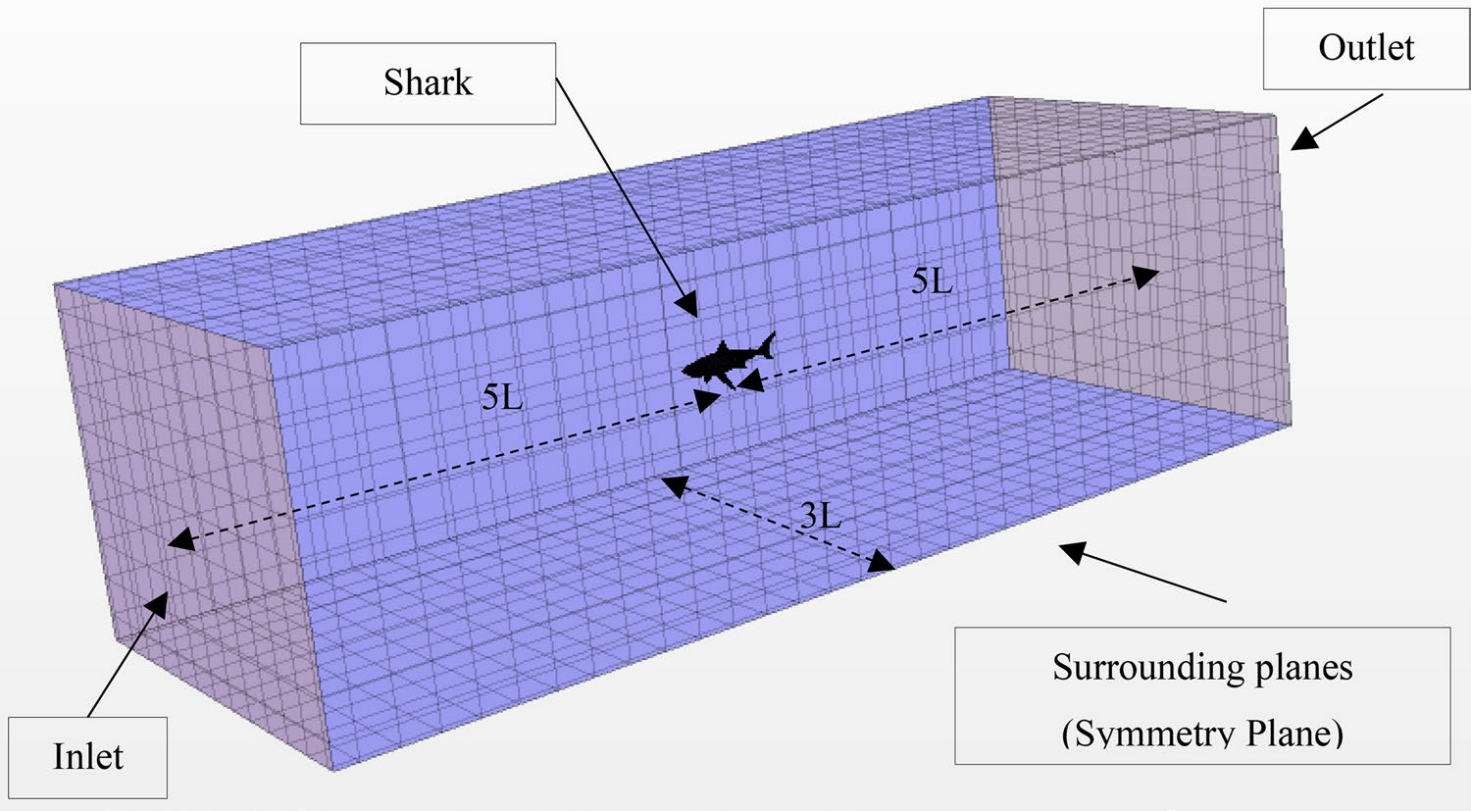
Domain and boundary conditions of the case 3 simulation (Created using Simcenter STAR-CCM + 2019.2.1 Build 14.04.013).

The computational domain is a cuboid, as shown in Fig. 5. According to ITTC Practical Guidelines for Ship CFD Applications guidelines^16^, the surrounding planes are defined as symmetry boundary conditions 1.5L from the body, while the shark and the remora models are set to non-slip wall conditions. The inlet is defined as the velocity inlet 5L upstream from the model. The outlet is positioned 5L downstream from the model and is defined as the pressure outlet. Additionally, the outlet was extended 3 times further, to verify the results and the analysis showed a negligible effect on the computed drag. To ensure that the width of the channel was adequate, the blockage ratios were computed, with the highest 0.33% appearing in the shark free-swimming condition which deems to be very low.

**Figure 6:**
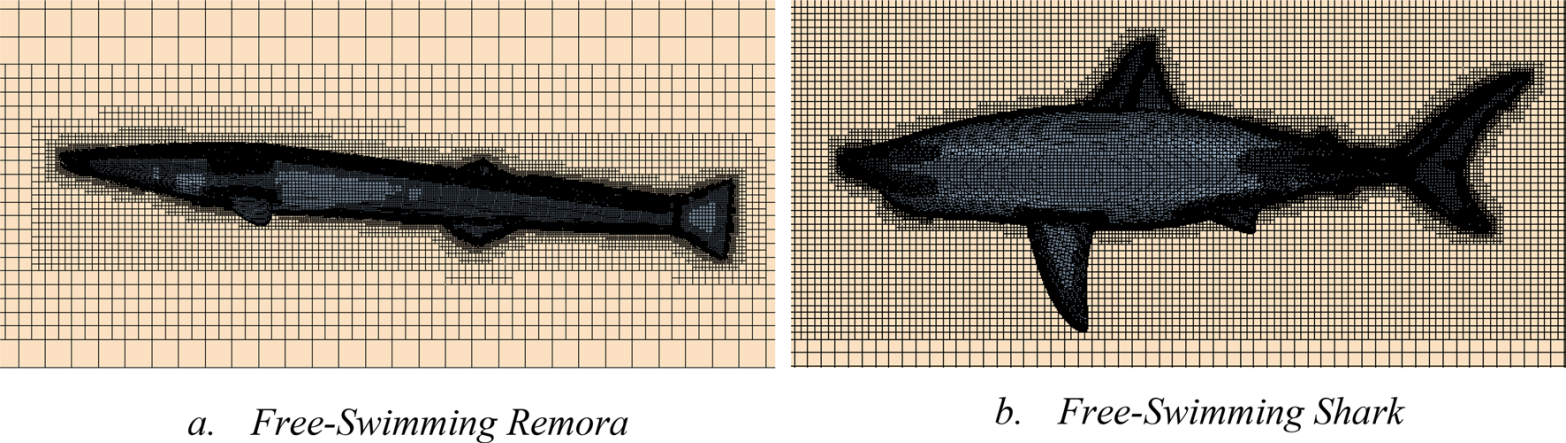
Mesh view for free-swimming simulations (Created using Simcenter STAR-CCM + 2019.2.1 Build 14.04.013).

The automatic meshing tool with volumetric control is used to generate the mesh. A more refined grid is used in the vicinity of the models which is about 0.1L. We note that this refinement was done in all the tested cases (free swimming, flat plate and attached) as indicated in Figs. 6, 11 and 20, respectively. The height of the first cell near the wall is controlled to have y + less than 5^15,17–19^. In total, around 3.4 million cells have been used for this simulation. The detailed mesh scenes for both simulations are presented in Fig. 6.

### Grid independence study

A grid independence study was conducted to verify the accuracy of the numerical simulation^20,21^. There are three different grid groups to be implemented in the CFD software STAR-CCM + namely: coarse, medium, and fine grid. A grid refinement ratio R = $\sqrt{2}$ is used. The simulation of the free-swimming remora is used to perform this study. The cell numbers of the three grids increased from 1.6 million cells for the coarse case to 8.4 million cells for the fine case (see Table 1). The numerical uncertainty values for the drag force is about 2.26%. Therefore, considering the computational cost, the medium grid will be used in the subsequent analysis.

**Table 1:** Grid independence study of free-swimming remora in 1 kn.

Grid	Grid count	Drag force (N)
Fine grid	8,446,216	1.07334E−01
Medium grid	3,724,865	1.10144E−01
Coarse grid	1,634,396	1.17083E−01

### Hydrodynamic performance of free-swimming remora and shark

This section shows the drag results of the remora and the shark in free-swimming conditions at different velocities. Nondimensional numbers are used to compare the resistance performance. The Reynolds number, *Re*, is calculated with following Eq. (1):

1 $$ Re = \frac{\rho *L*V}{\mu }, $$

where *ρ* is the density of the water, 997 kg/m^3^; *L* is the reference length, m; *V* is the incoming velocity, m/s; *µ* is the dynamic viscosity of water, 0.001 kg/(ms).

And the drag coefficient, $C_{D}$, is calculated with following Eq. (2):

2 $$ C_{D} = \frac{F}{{\frac{1}{2} * \rho * V^{2 } * A}}, $$

where *F* is the drag force, N; *A* is the reference frontal area of the model (width * height, m^2^).

**Figure 7:**
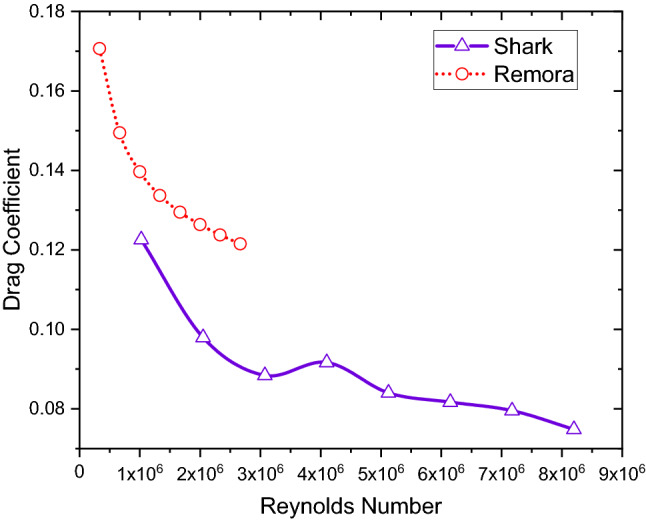
Drag coefficients vs. Reynolds numbers of remora and shark in free swimming.

The simulation results are shown in Table 2, whilst the drag coefficients are shown in Fig. 7. In the table, it can be seen that at the same $V$, the remora fish has much lower Reynolds number than the shark due to the different body lengths. However, under the same Reynolds number as shown in Fig. 7, the drag coefficient of the remora is much higher than the one of the shark. Therefore, in its free-swimming mode, the remora fish does not show any competitive drag performance. In fact, the remora has a non-streamlined body compared to the highly streamlined body of the shark body. We observe that the remora body has a flat suction pad on top of the head, which could make its body less hydrodynamically efficient. Additionally, we also observe in Fig. 7, that at 4 knots (Reynolds number of 4.5 × 10^6^), the shark resistance characteristics indicate laminar to turbulent transition, due to a peak in the drag coefficient. At the investigated is velocity range (up to Reynolds number of 1.3 × 10^6^), flow transition is not observed on the remora, indicating fully turbulent flow around the remora.

**Table 2:** The drag results of case 2 in various velocities.

Velocity	Velocity	Free Swimming Remora			Free Swimming Shark		
		Re	Drag	C_D_	Re	Drag	C_D_
(kn)	(m/s)		(N)			(N)	
1	0.514	3.331E+05	0.110	0.17068	1.025E+06	2.329	0.12257
2	1.028	6.662E+05	0.386	0.14946	2.050E+06	7.445	0.09794
3	1.542	9.993E+05	0.811	0.13967	3.075E+06	15.119	0.08839
4	2.056	1.332E+06	1.380	0.13369	4.100E+06	27.875	0.09167
5	2.570	1.665E+06	2.089	0.12949	5.125E+06	39.914	0.08401
6	3.084	1.999E+06	2.936	0.12637	6.149E+06	55.872	0.08166
7	3.598	2.332E+06	3.913	0.12376	7.174E+06	74.040	0.07951
8	4.112	2.665E+06	5.019	0.12153	8.199E+06	90.989	0.07481

**Figure 8:**
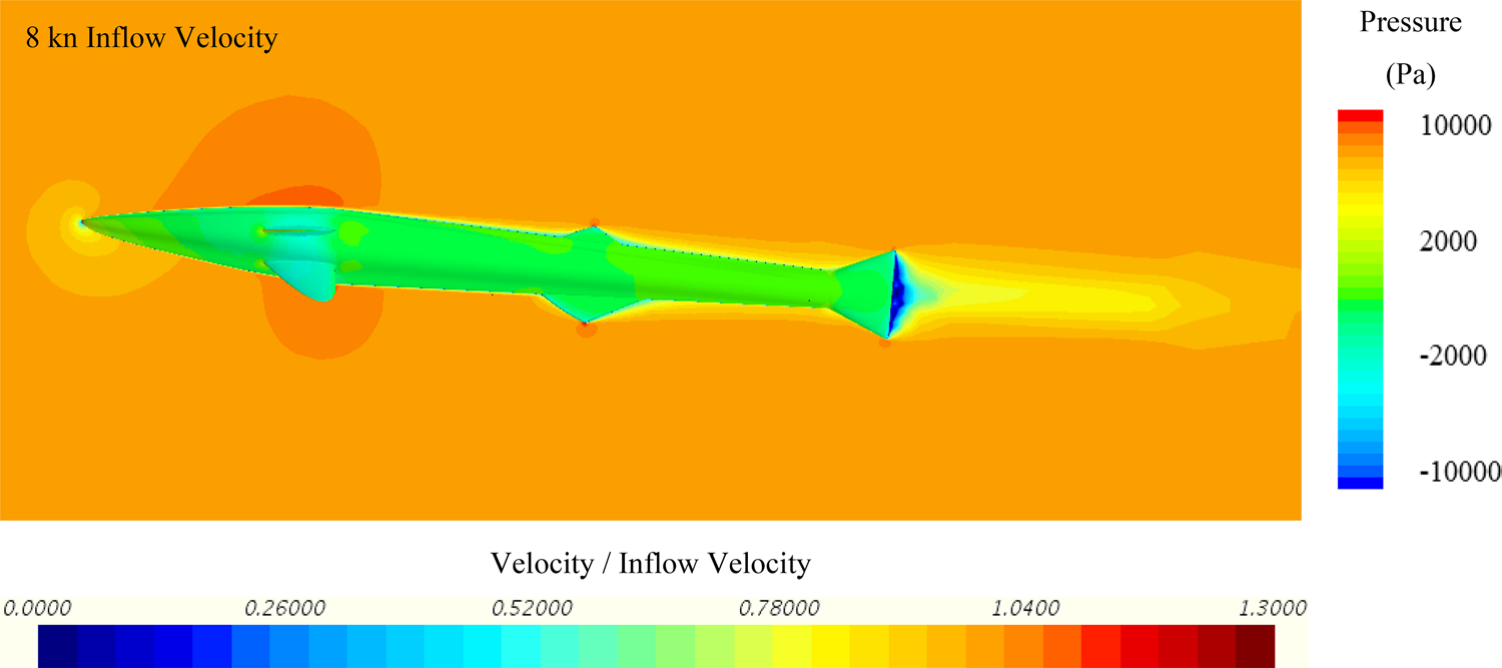
Velocity and pressure contours of the remora fish in free-swimming condition (Pressure contour displayed on the surface of model, and velocity contours displayed on the section plane) (Created using Simcenter STAR-CCM + 2019.2.1 Build 14.04.013).

**Figure 9:**
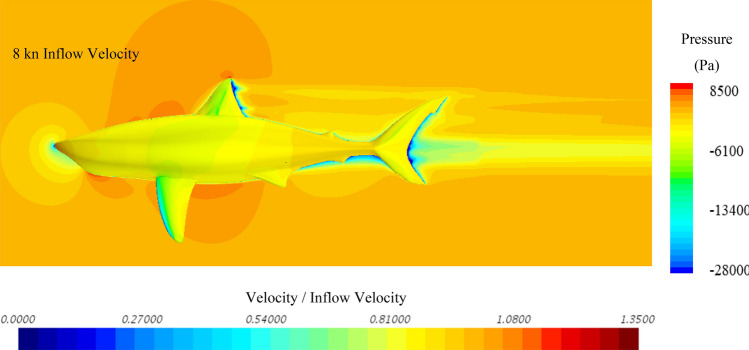
Velocity and pressure contours of the shark in free-swimming condition (Pressure contour displayed on the surface of model, and velocity contours displayed on the section plane) (Created using Simcenter STAR-CCM + 2019.2.1 Build 14.04.013).

To further investigate the hydrodynamic characteristics of both remora and shark in free-swimming modes, velocity contours in the mid-section and pressure contours on the body have been extracted, and because the contours between varying velocities are similar, the 8kn inflow velocity cases for the remora and the shark are displayed in Fig. 8 and Fig. 9. It is worthy noticing that as shown in Fig. 9 due to the bulky build of the whitetip shark, adverse pressure gradient regions with low flow velocity develop gradually after the belly and the back regions. This hints the hydrodynamic reasons why the remora prefers to attach to those locations rather than to the pectoral fins.

## Effect of developed boundary layer on the remora fish swimming

Once the free-swimming conditions of both remora and shark have been evaluated, this section focuses on investigating the hydrodynamic impact of the boundary layer flow on the remora fish swimming performance. To investigate this question, a fundamental approach of a boundary layer flow over a flat plate has been used. We demonstrate this in the following section.

### Systematic approach to introduce the boundary layer flow

In this section, we set to investigate firstly, the effect of the boundary layer thickness in the drag reduction rate of the remora. Secondly, we attempt to develop a relationship between the average velocity of a boundary layer profile to the drag reduction on the remora. These two points will help in future design problems where fast design computations could be required. To address the first point, we systematically vary the flow velocity and the remora’s attachment location to a flat plate. This will provide us with a relationship that computes the drag reduction rate to the boundary layer height in which the remora is swimming. To address the second point, we estimate first a drag reduction rate based on the average velocity of the boundary layer and compare this estimation to the drag reduction rate computed with the simulations.

By defining a reference frame at the leading-edge and at the centre of a flat plate, with the $x$-axis oriented in the streamwise direction, the transition point ${x}_{c}$_,_ is the $x$-coordinate where laminar to turbulent transition occurs. This transition point ${x}_{c,}$ is estimated using Eq. (3) and the results are shown in Table 3.

3 $$  x_{c}  = \frac{{{\text{Re}}*~{\upmu }}}{{\rho *V}}  $$

**Table 3:** Estimated Transition Point ${\mathrm{x}}_{\mathrm{c}}$ with varying flow velocity V.

V, kn	V, m/s	${x}_{c}$, m
1	0.514	0.097493
2	1.028	0.048747
3	1.542	0.032498
4	2.056	0.024373

Four different locations downstream from the leading edge and along the flat plate (10 m, 20 m, 30 m and 40 m) are chosen to perform the systematic simulation. They are all in the turbulent flow regime, as indicated by ${x}_{c}$ in Table 3. Therefore, a turbulent boundary layer flow is expected and to estimate the boundary layer thickness δ, Eq. (4) is used. In the Eq. (4), $L$, is the distance between the attachment location and the leading edge of the flat plate^22^.

4 $$ \delta = \frac{0.37*L}{{Re^{\frac{1}{5}} }} $$

It can be seen in Table 4 that with the varying velocities and the attachment locations, the developed boundary layer thickness varies. We note that the ratio between the boundary layer thickness and the height of the remora fish ($\delta$/H) ranges from 127.58 to 510%, and that slower velocities provide thicker boundary layers.

**Table 4:** Thickness of flat-plate boundary layer.

L, m	V, kn	δ, m	H, m	δ/H (%)
Length from the leading edge of the flat plate	Inflow velocity	Thickness of boundary layer	Height of the remora	The ratio between δ and H
10	1	0.1683	0.1	168.35
	2	0.1466		146.55
	3	0.1351		135.14
	4	0.1276		127.58
20	1	0.2931		293.11
	2	0.2552		255.16
	3	0.2353		235.29
	4	0.2221		222.13
30	1	0.4054		405.41
	2	0.3529		352.93
	3	0.3254		325.44
	4	0.3072		307.25
40	1	0.5103		510.33
	2	0.4443		444.27
	3	0.4097		409.66
	4	0.3868		386.76

### CFD simulation methodology

With the above estimation, this section simulates the remora swimming in the varying boundary layer thicknesses previously obtained and computes the drag on the remora, when covered by the flat plate boundary layer flow. In total 16 simulations were conducted with four remora attachment locations downstream of the leading edge of the flat plate (10 m, 20 m, 30 m, and 40 m) at four velocities, 1kn, 2kn, 3kn, and 4kn.

**Figure 10:**
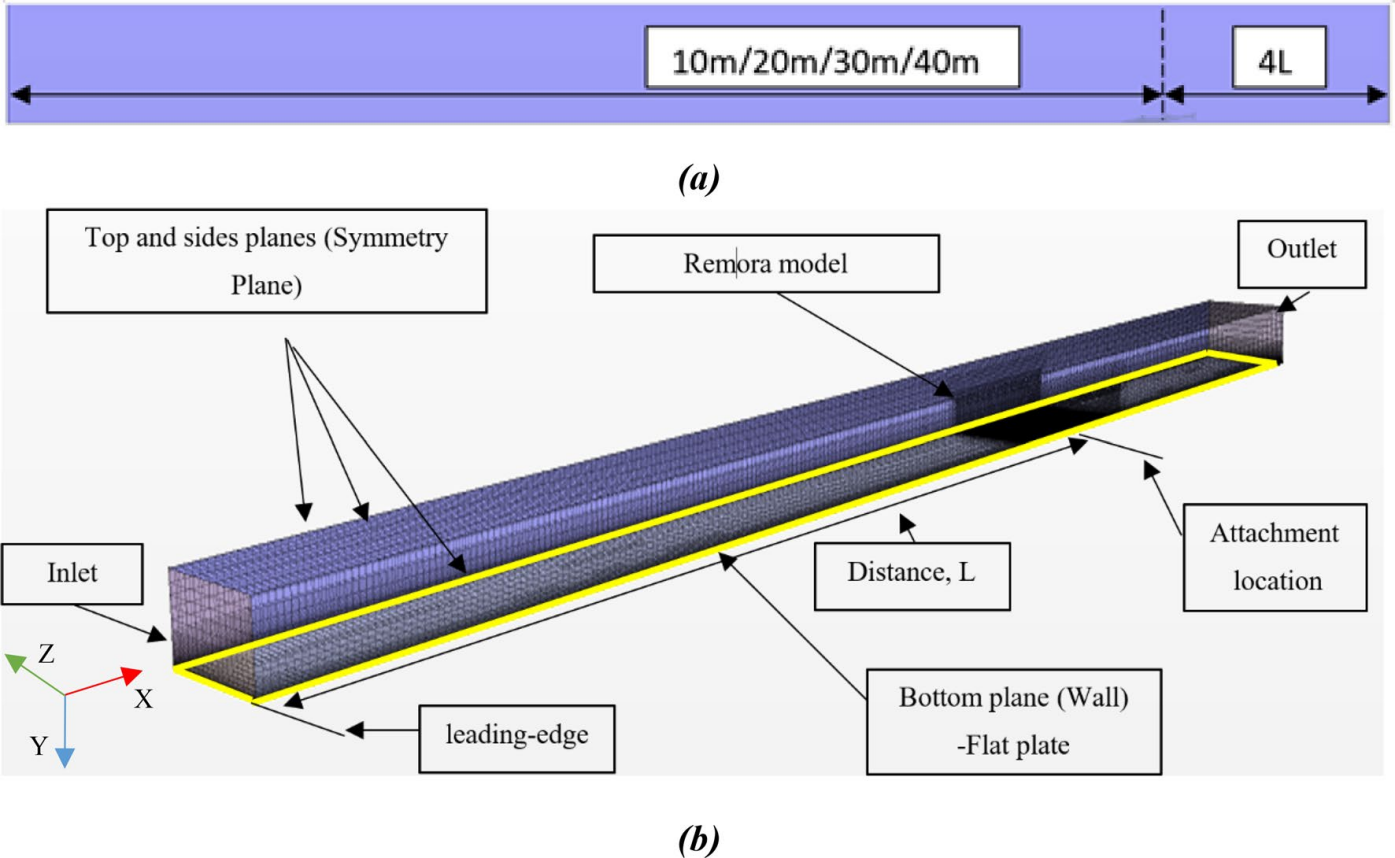
Domain and boundary conditions of the flat plate simulation, a) side view and b) three-dimensional view of the domain (Created using Simcenter STAR-CCM + 2019.2.1 Build 14.04.013).

In this case, the computational domain is also a cuboid domain. The boundary conditions are shown in Fig. 10. The pressure outlet boundary is positioned 4L downstream of the remora model. The upstream boundary is defined as velocity inlet. Its distance is varied between 10 m, 20 m, 30 m, and 40 m upstream from the remora fish (see Fig. 10a). This is to introduce different the boundary layer thicknesses. The remora model and the flat plate are set as non-slip wall conditions. The height of the domain is 2L and the distance between the two side planes is 2L. The top and sides planes are specified as symmetry planes. The boundary conditions and the full length of the flat plate are shown in Fig. 10b. In the figure, the velocity inlet boundary is at 10 m. The remora is attached to the bottom plane.

The size of the domain was defined according to the ITTC Practical Guidelines for Ship CFD Applications^16^. The surrounding boundaries are located between 1 to 2 full lengths away from the model. The blockage ratio is very low as well, 0.25% in this simulation. The downstream boundary is positioned 4 full lengths away from the model and it was also elongated 3 times to verify the results, which showed a negligible effect on the computed drag.

**Figure 11:**
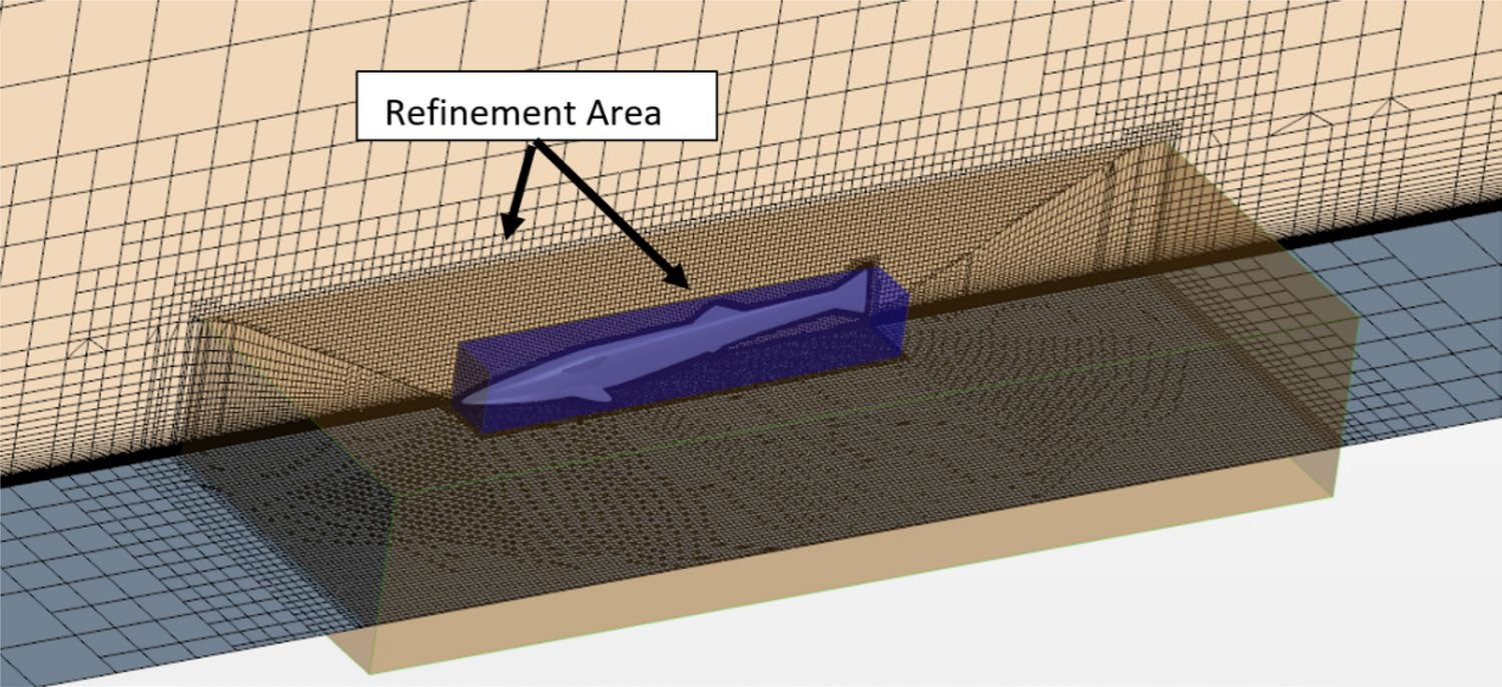
The mesh around the remora fish when attached onto the flat plate (Created using Simcenter STAR-CCM + 2019.2.1 Build 14.04.013).

The mesh of the remora model is generated by the trimmer mesh in STAR-CCM +. As shown in Fig. 11, volumetric refinement has been applied in the vicinity of the remora fish. Meanwhile, to capture the boundary layer development of the bottom plane, 80 layers of prism mesh are used on the bottom plane. We note that the y + is kept below 5 in all of the simulations, however, in the flat plate y + is below 1.

### Result analysis and discussion about the boundary layer effect

**Figure 12:**
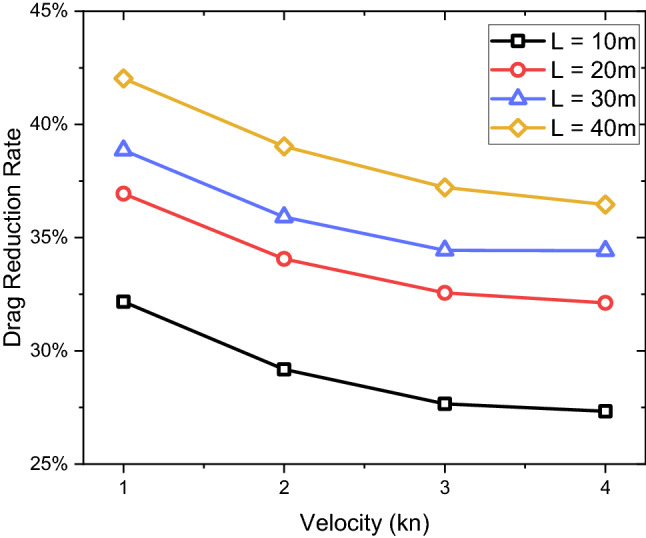
The drag reduction rate vs. the incoming velocity as attached on different locations from the leading edge of the flat plate.

With the above setup, the drag characteristics can be achieved over varying boundary layer thicknesses. As summarised in Table 5, it can be seen that when the remora fish is swimming in the boundary layer a drag reduction can be achieved, ranging between 27 to 42%. This drag reduction shows direct relationship with the boundary layer thickness which is maximum at the lowest speed ($V$=1kn) with the longest distance ($L$=40 m) as indicated in Table 5 and Fig. 12. Therefore, from a drag reduction point of view, the remora fish tends to choose areas with a developed boundary layer to minimise the resistance on its body.

**Table 5:** Drag reduction rate of the remora fish swimming in the boundary layer for different flat plate lengths, velocities, boundary layer thicknesses and boundary layer to remora height ratios.

Length of flat plate	Velocity	Re	δ	δ/H (%)	Boundary layer—drag	Free swimming—drag	Drag reduction rate (%)
(m)	(kn)	Flat plate	(m)		(N)	(N)	
10	1	5,128,568	0.1683	168.35	0.07471	0.11014	32.17
	2	10,257,136	0.1466	146.55	0.27322	0.38580	29.18
	3	15,385,704	0.1351	135.14	0.58682	0.81118	27.66
	4	20,514,272	0.1276	127.58	1.00320	1.38040	27.33
20	1	10,257,136	0.2931	293.11	0.06945	0.11014	36.94
	2	20,514,272	0.2552	255.16	0.25441	0.38580	34.06
	3	30,771,408	0.2353	235.29	0.54708	0.81118	32.56
	4	41,028,544	0.2221	222.13	0.93700	1.38040	32.12
30	1	15,385,704	0.4054	405.41	0.06735	0.11014	38.86
	2	30,771,408	0.3529	352.93	0.24726	0.38580	35.91
	3	46,157,112	0.3254	325.44	0.53185	0.81118	34.44
	4	61,542,816	0.3072	307.25	0.90525	1.38040	34.42
40	1	20,514,272	0.5103	510.33	0.06385	0.11014	42.03
	2	41,028,544	0.4443	444.27	0.23520	0.38580	39.03
	3	61,542,816	0.4097	409.66	0.50924	0.81118	37.22
	4	82,057,088	0.3868	386.76	0.87716	1.38040	36.46

**Figure 13:**
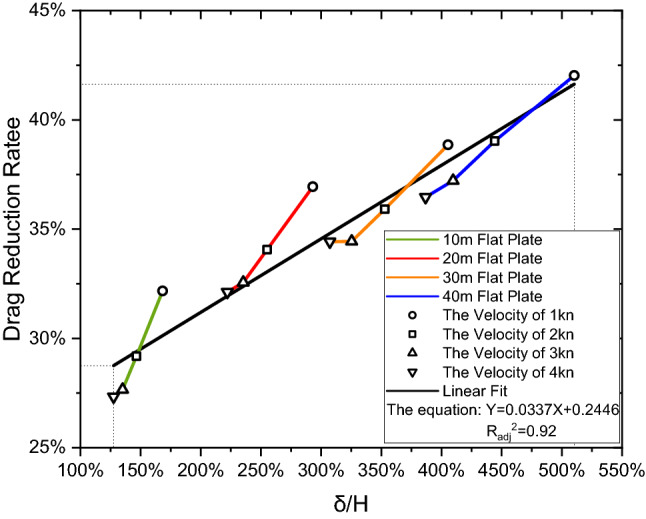
Linear fitting for the drag reduction rate versus boundary layer thickness to remora height ratio.

Figure 13 shows the drag reduction rate (${D}_{r}$) versus $\delta /H$. The relationship is roughly linear. Hence the drag reduction rate can be estimated with the following Eq. (5):

5 $$ D_{r} = 0.0337\frac{\delta }{H} + 0.2446 $$

We utilised the adjusted coefficient of determination (${{R}_{adj}}^{2}$) as an indicator to measure the linearity of the data. A high value indicates a linear relationship. The adjusted coefficient of determination ${{R}_{adj}}^{2}$ restrains the effect of the number of independent variables and its definition is given by:

6 $$ R_{adj}^{2} = 1 - \left( {1 - R^{2} } \right)\frac{n - 1}{{n - p - 1}} $$

where $R$ is the coefficient of determination, *n* is the sample size, and *p* is the number of independent variables. Here, we computed ${{R}_{adj}}^{2}$ to be 0.92.

**Figure 14:**
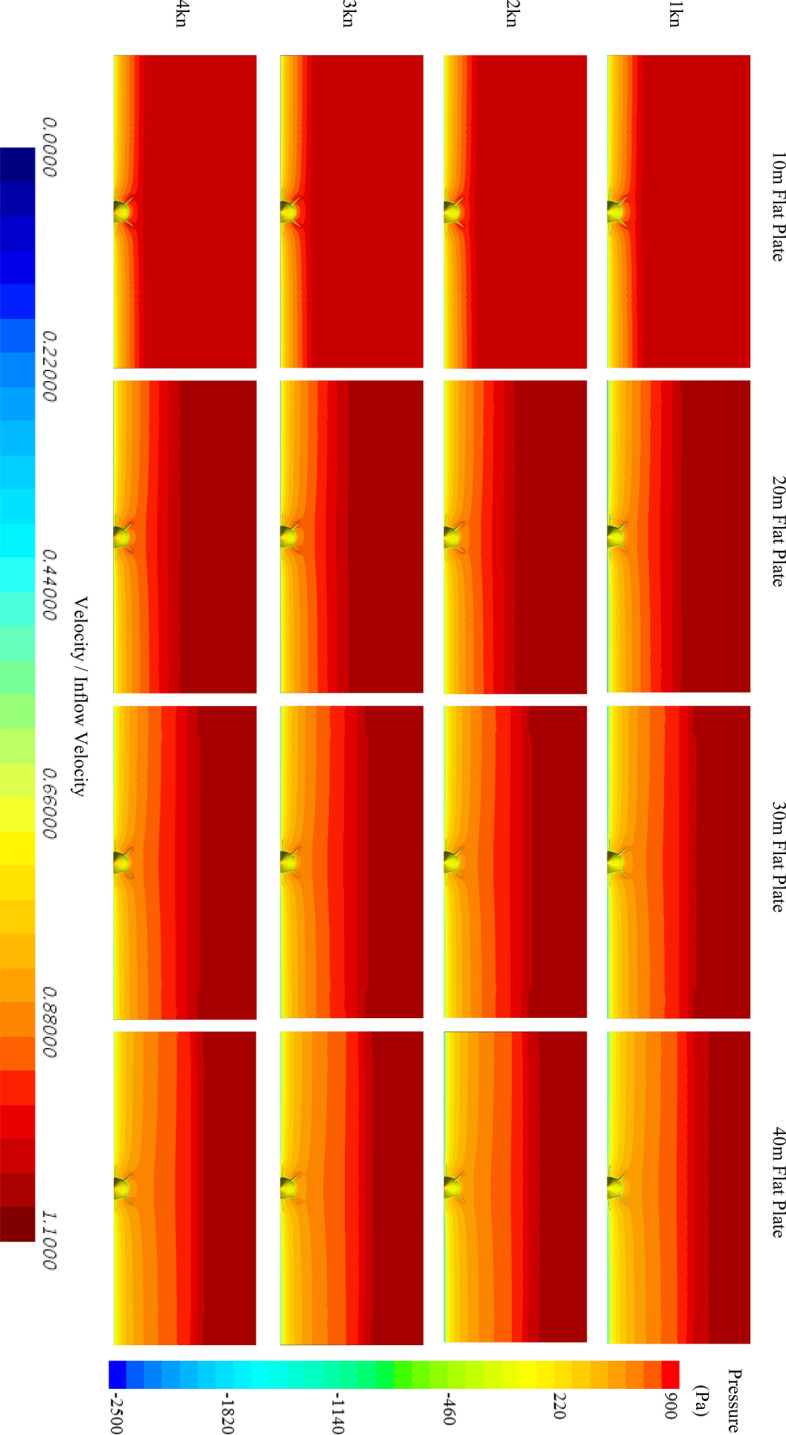
The velocity contour on the cross section and pressure contour on the remora fish (Pressure contour displayed on the surface of model, and velocity contours displayed on the section plane) (Created using Simcenter STAR-CCM + 2019.2.1 Build 14.04.013).

**Figure 15:**
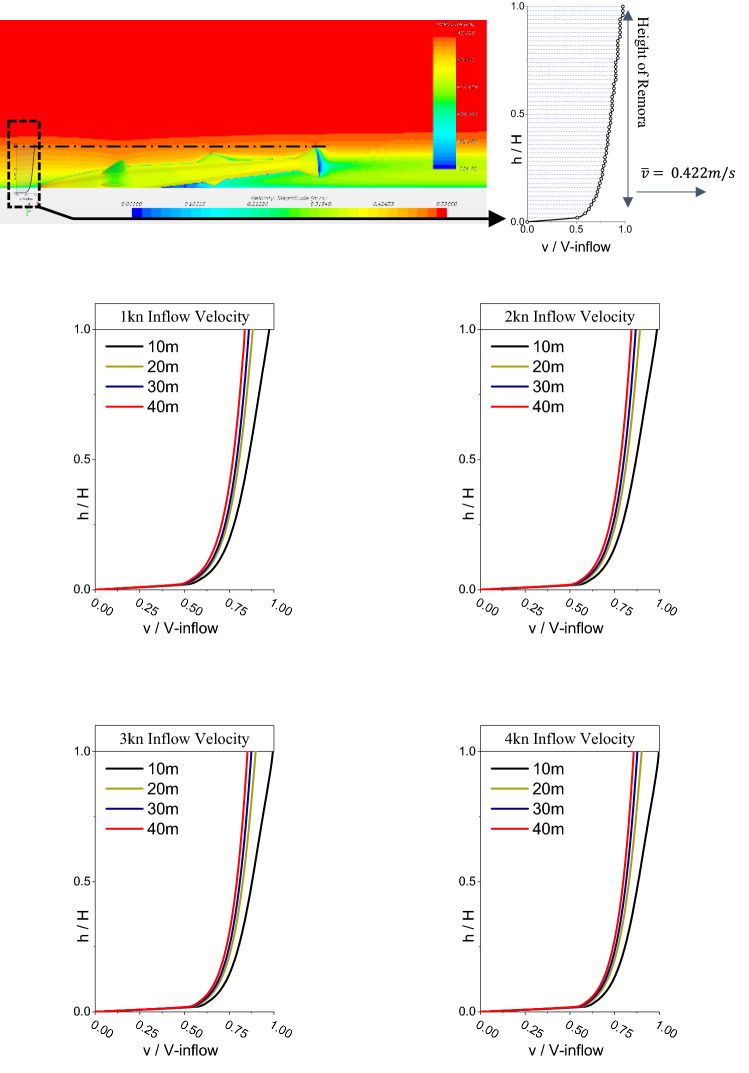
Summary of the velocity distributions in boundary layer at different velocities within the height of the remora (H).

On the other hand, as shown in Fig. 14, the velocity distribution and pressure contours are summarised and it can be seen that the boundary layer thickness increases with increasing $L$ and decreases with increasing $V$. For a detailed comparison, Fig. 15 shows a summary of the boundary layer profiles of the tested cases at the different flow velocities. From this figure, the average velocity of the boundary layer within the height of remora (H) can be extracted. By averaging the flow velocity of the boundary layers (${V}_{average}$) and considering thincoming velocity upstream of the remora (${V}_{incoming}$), the estimated drag reduction rate (${D}_{r}^{e}$) is shown below in Eq. (7):

7 $$ D_{r}^{e} = \frac{{v_{average}^{2} - v_{incoming}^{2} }}{{v_{incoming}^{2} }}. $$

**Figure 16:**
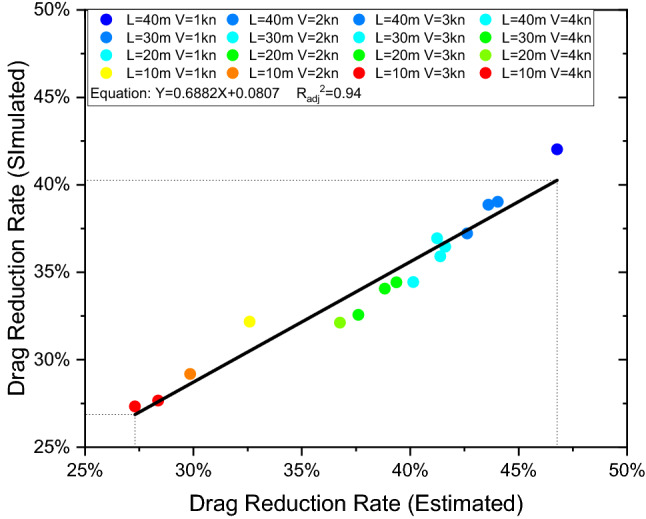
The linear fitting equation for the drag reduction rate between the simulated one and the estimated one using the average velocity.

As shown in Fig. 16, a linear relationship between the simulated drag reduction rate and the estimated one using the average velocity can be derived with the following Eq. (8) with ${{R}_{adj}}^{2}$=0.94:

8 $$ Y = 0.6882X + 0.0807 $$

Equation (8) is derived from the linear relationship of Fig. 16. In the figure, *X* is the estimated drag reduction rate, which is computed by Eq. (7), while *Y* is the drag reduction rate resulting from our simulations. We believe that Eqs. (5) and (8) are helpful as a reference to estimate the drag reduction rate in the boundary layer for future designs.

A cross validation is presented versus the law of the wall of the flat plate boundary layer. Through the results of the flat plate boundary layer simulation, the relationship between dimensionless velocity, *u*^+^, and wall coordinate, *y*^+^, can be extracted and compared with the law of the wall of the flat plate boundary layer. Moreover, the dimensionless velocity, *u*^+^, and wall coordinate, *y*^+^, can be calculated by the following Eqs. (9), (10) and (11)^23^:

9 $$ u_{\tau } = \sqrt {\frac{{\tau_{w} }}{\rho }} , $$

10 $$ y^{ + } = \frac{{y*u_{\tau } }}{v}, $$

11 $$ u^{ + } = \frac{u}{{u_{\tau } }}, $$

where ${{\varvec{u}}}_{{\varvec{\tau}}}$ is the friction velocity, m/s; ${{\varvec{\tau}}}_{{\varvec{w}}}$ is the wall shear stress, Pa; ${\varvec{\rho}}$ is the density of the water; ${\varvec{y}}$ is the distance from the wall, m; ${\varvec{v}}$, is the kinematic viscosity, m^2^/s; ${\varvec{u}}$ is the velocity, m/s.

**Figure 17:**
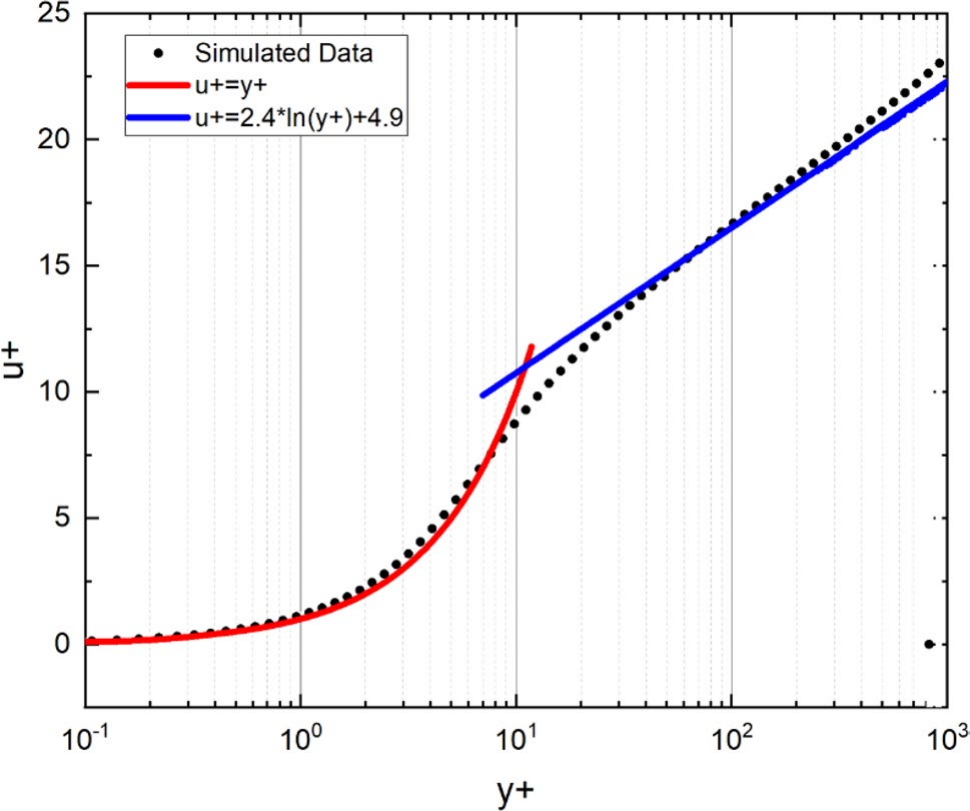
Dimensionless velocity, u + , vs. wall coordinate, y +.

The results are plotted in Fig. 17. The simulation results show good agreement with the law of the wall of the flat plate boundary layer.

## Effect of attachment locations on the remora fish swimming

### Model setup of the attachment location investigation

**Figure 18:**
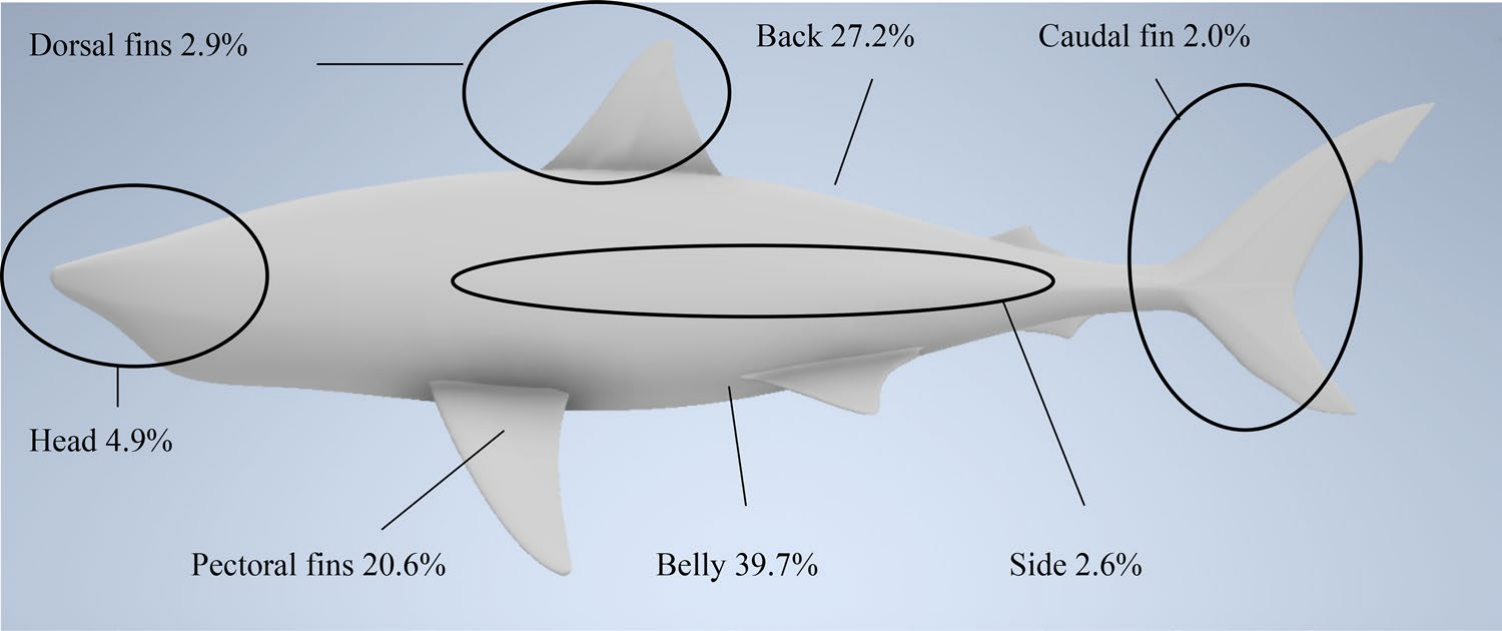
The probability that the remora attaches to the surface of shark at various locations (reproduced based on^8^).

**Figure 19:**
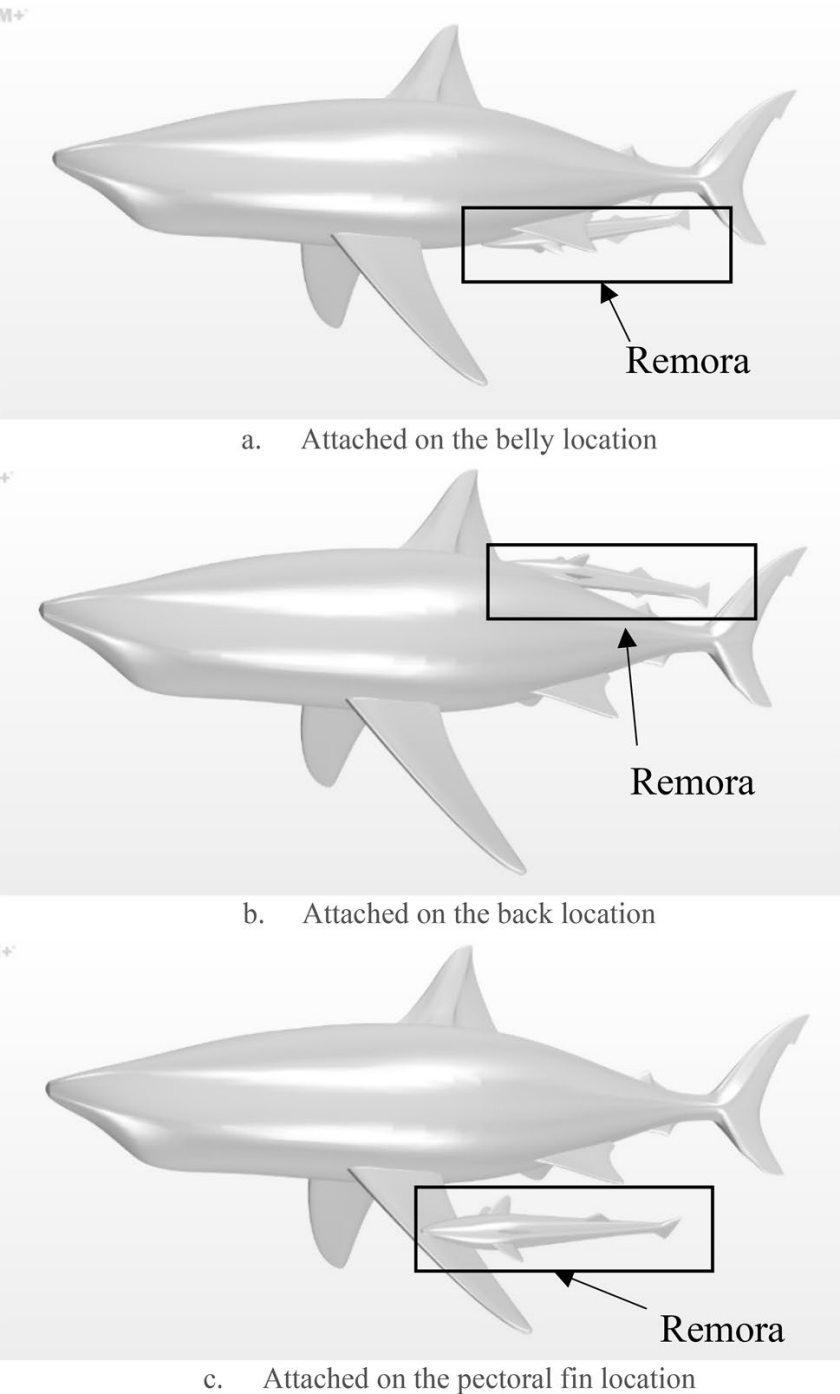
Attachment locations of the remora fish in the (**a**) belly, (**b**) back and (**c**) pectoral fin of the shark (Created using Simcenter STAR-CCM + 2019.2.1 Build 14.04.013).

Based on the previous section, it is found that taking the advantage of the developed boundary layer the drag experienced by the remora fish is significantly reduced. The following section explores the effect of the attachment locations of the remora fish on the body of the shark. According to the study conducted by Brunnschweiler et al. in 2006, three favourable attachment locations (the belly, the back and the pectoral fin, as reproduced in Fig. 18) were identified and they are the ones investigated in this study. The CFD models were created, including the remora attached to the shark at the different locations, as shown in Fig. 19.

### CFD simulation methodology

**Figure 20:**
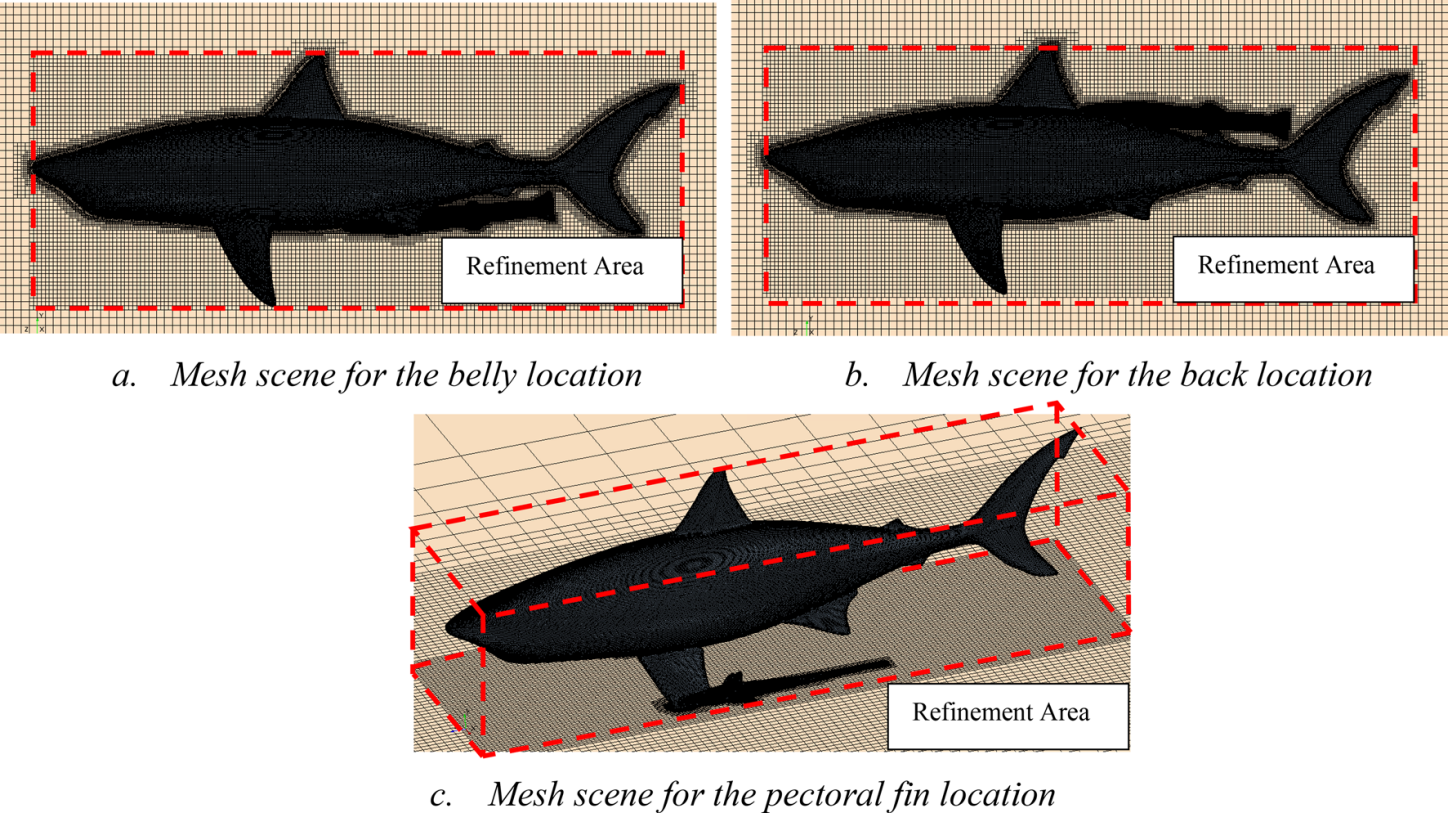
Different meshing scenarios with the remora fish attached to the shark (**a**) at the belly, (**b**) at the back and (**c**) at the pectora fin (Created using Simcenter STAR-CCM + 2019.2.1 Build 14.04.013).

The boundary conditions of the computational domain for the belly, back and pectoral fin cases, are specified in the same manner as those of Sec. 3. Nine different speeds are examined. First, speeds over the range between 1 to 8 knots, in intervals of 1knot, and then, the average speed of oceanic whitetip shark (1.3 knots). The mesh details and refinement areas are shown in Fig. 20.

### Result analysis and discussion about the attachment locations

The drag characteristics of the remora when attached to different locations of the shark are computed. By comparing the results to the equivalent free-swimming conditions, the drag reduction rate for the remora, and the drag increase rate for the shark are determined.

**Figure 21:**
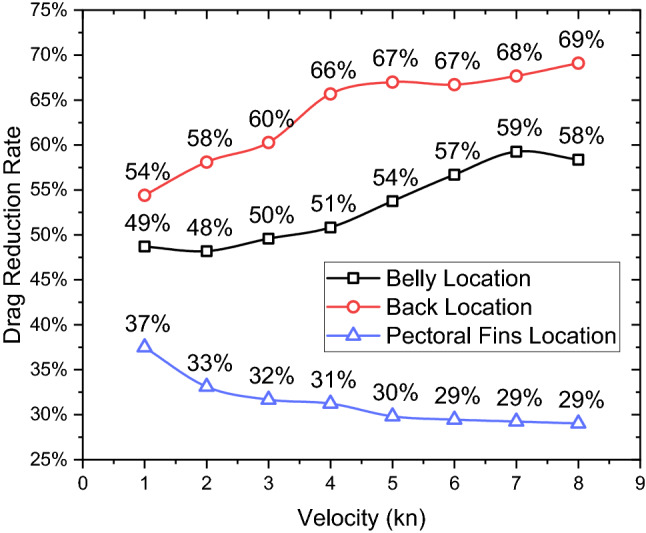
The rate of the drag reduction in a range of velocities when the remora attached on different locations.

Figure 21 presents the trends of the drag reduction rate of the remora when attached to different locations. It can be noted that for the belly and the back locations, the drag reduction rates of the remora increase with the rise of the velocity, from 49 to 59% and 54 to 69% respectively. On the contrary, for the pectoral fins location, the drag reduction rate decreases from 37 to 29% with increasing velocity, and the drag reduction rate seems to converge towards a constant value after 4kn. In summary, the belly attached case and the back attached case have higher rate of drag reduction, with the back attached case showing the highest reduction.

**Figure 22:**
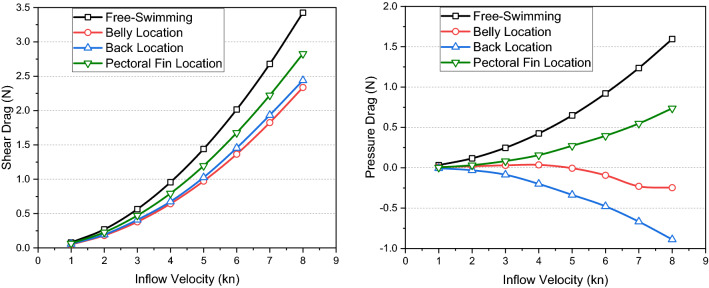
Comparations of the pressure forces and the shear forces for remora in different conditions.

The drag components of the remora, pressure drag and shear drag, are shown in Fig. 22, for the free-swimming condition and the different attachment locations versus different velocities. With increasing velocity, the trends of the shear drag are similar for all four conditions. Regarding to the pressure drag, the free-swimming and pectoral fins-attachment conditions have a similar trend. On the contrary, for the belly and back attachment conditions the pressure drag points to the opposite direction and provide a drag reduction for the remora due to the adverse pressure gradient. It should be noted that at the back location there is a dorsal fin in front of remora and the adverse pressure gradient region appears to be built up behind the dorsal fin, therefore the pressure force become negative pointing opposite to the direction of the shear drag and results in the maximum drag reduction for the remora. Similarly, when the remora attaches to the belly of the shark, the incoming flow is largely blocked by the shark body, and after 4 kn the forward thrust provided by pressure force increases. Whereas, when attached onto the pectoral fin, the remora is exposed to the incoming flow, hence creating the lowest drag reduction rate.

**Figure 23:**
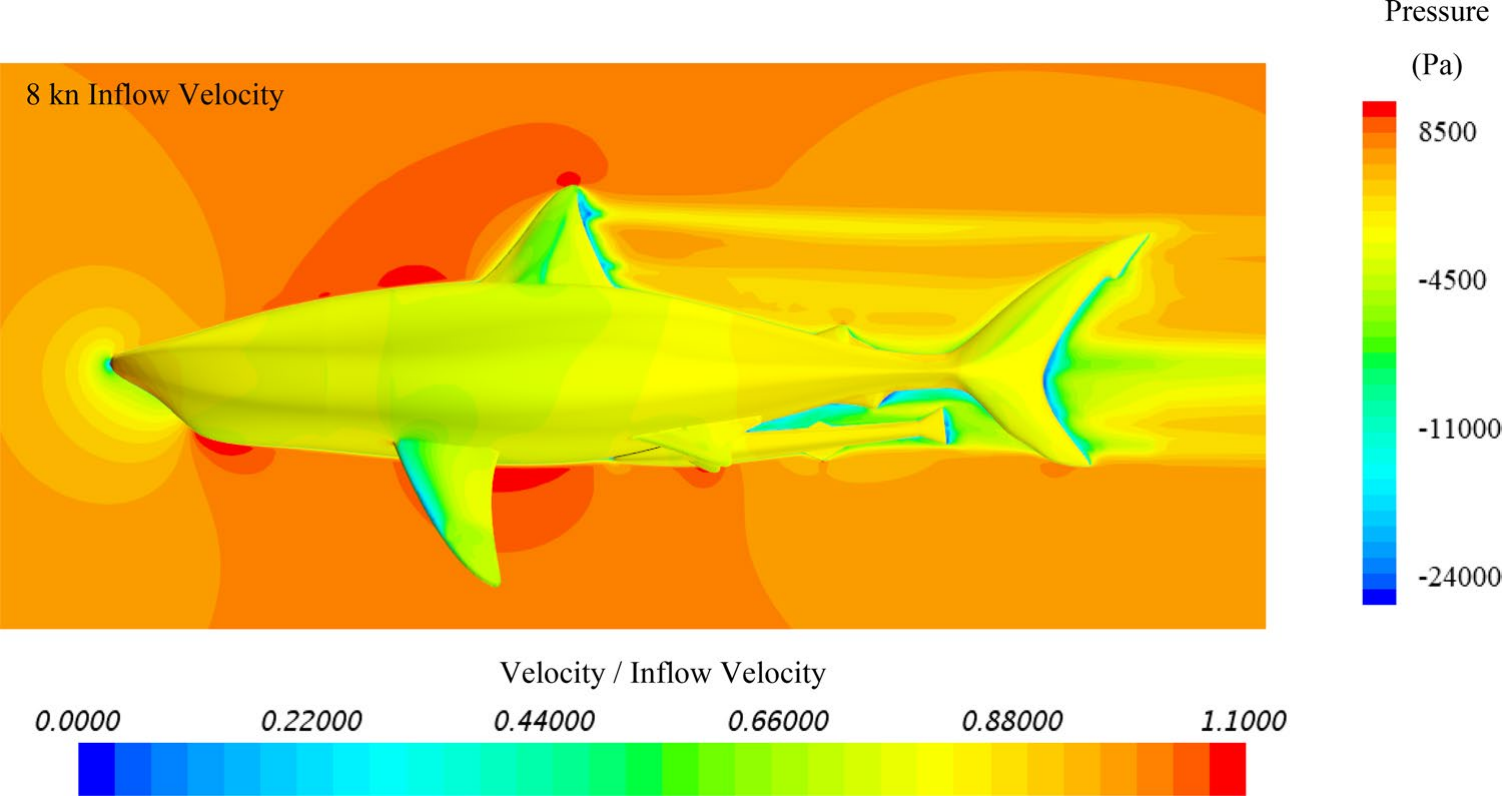
Velocity and pressure contours of the remora attached to the belly of the shark (Pressure contour displayed on the surface of model, and velocity contours displayed on the section plane) (Created using Simcenter STAR-CCM + 2019.2.1 Build 14.04.013).

**Figure 24:**
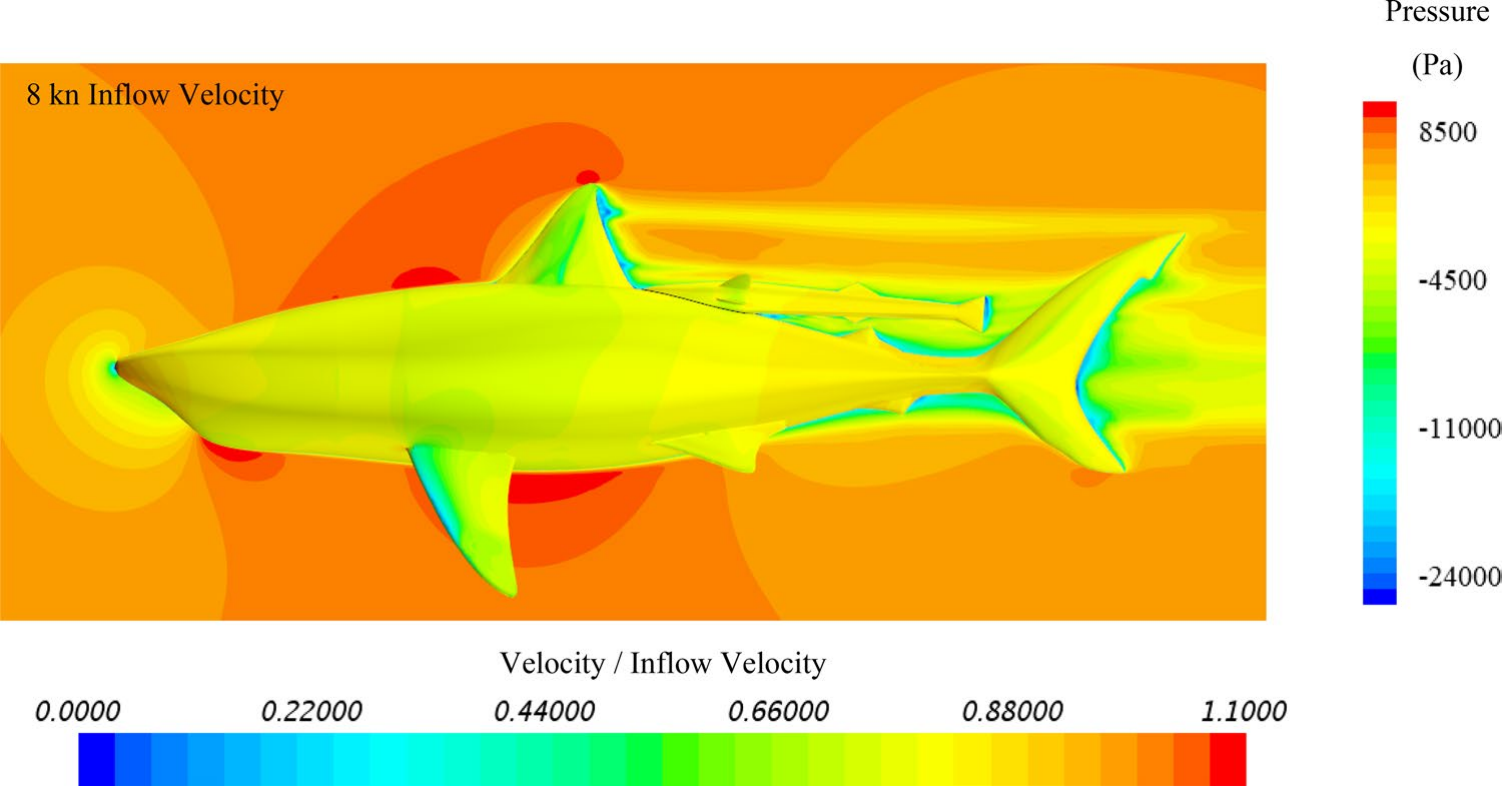
Velocity and pressure contours of the remora attached to the back of the shark (Pressure contour displayed on the surface of model, and velocity contours displayed on the section plane) (Created using Simcenter STAR-CCM + 2019.2.1 Build 14.04.013).

**Figure 25:**
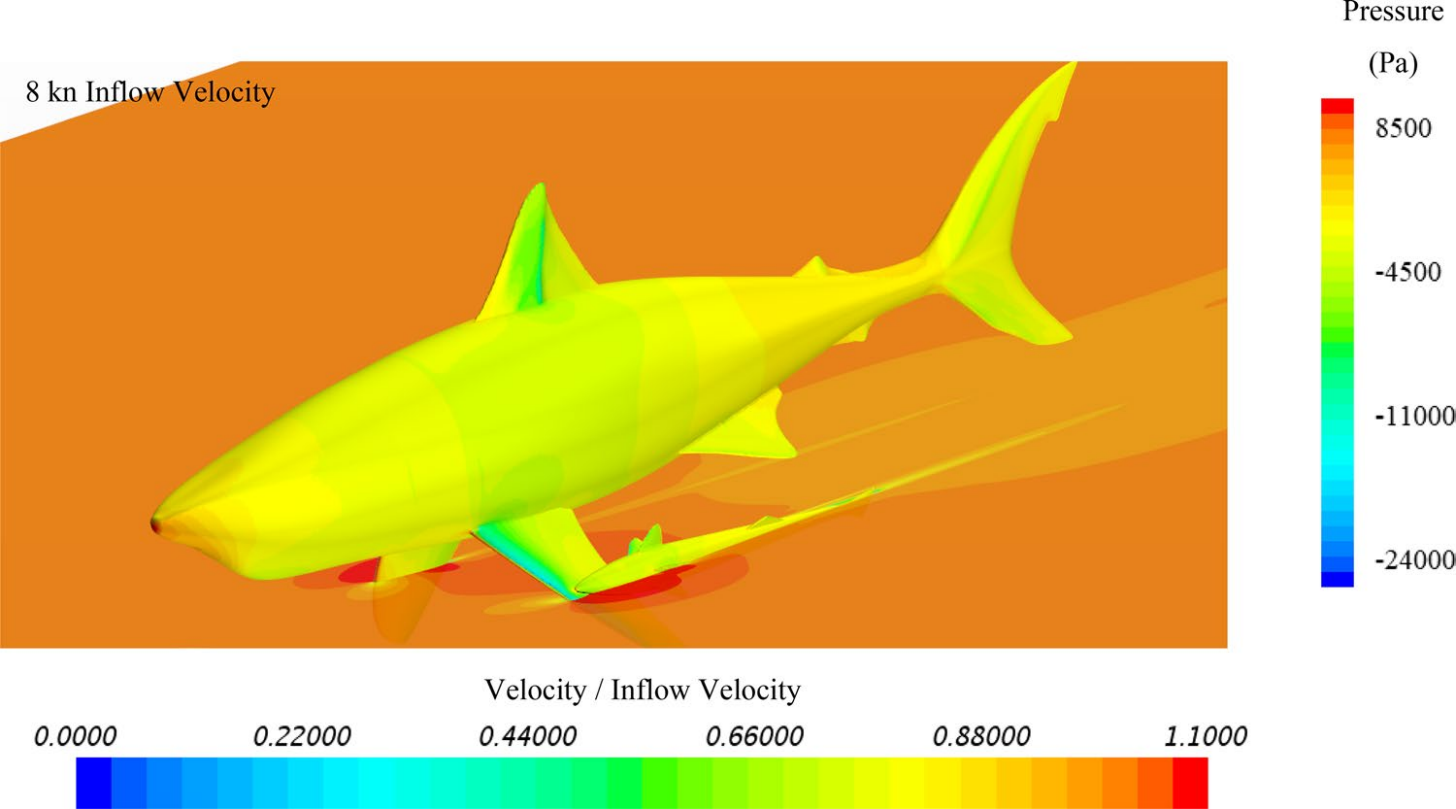
Velocity and pressure contours of the remora attached to the pectoral fin of the shark (Pressure contour displayed on the surface of model, and velocity contours displayed on the section plane) (Created using Simcenter STAR-CCM + 2019.2.1 Build 14.04.013).

As for the belly-location, the back-location and the pectoral fin-location cases, the simulated results are plotted with the pressure on the surface and the velocity on the section, as shown in Figs. 23, 24 and 25. In the belly location case presented in Fig. 23, the area around the remora has displayed as a low velocity region, in which remora is hiding in this area. In this area, adverse pressure gradient builds up because of the blockage of the shark body. Therefore, the shear component of the drag presented the highest reduction comparing to other attachment locations. And after 4kn velocity the pressure force provide a forward thrust to remora, which demonstrated the effect by adverse pressure gradient.

Regarding to back attached case, in Fig. 24, a larger adverse pressure gradient region builds up behind the dorsal fin because of slope of the shark back, thus the pressure force provides a higher forward thrust than the one in the belly attached case. Therefore, it can be confirmed that higher drag reduction rates for remoras are related to the low velocity and the adverse pressure gradient regions.

However, for the pectoral-fin attached case, although the pectoral fin of shark blocks a part of incoming flow, the most of remora body is exposed to the incoming flow and high-velocity and high-pressure regions can be found around the remora body. Therefore, in this attachment location the drag reduction rate is the lowest. Meanwhile from the trends of pressure force in Fig. 22 the pressure force increases with the flow speed showing similar trend with the free-swimming condition, which proofs that no adverse pressure gradient has acted on the remora during this condition.

**Figure 26:**
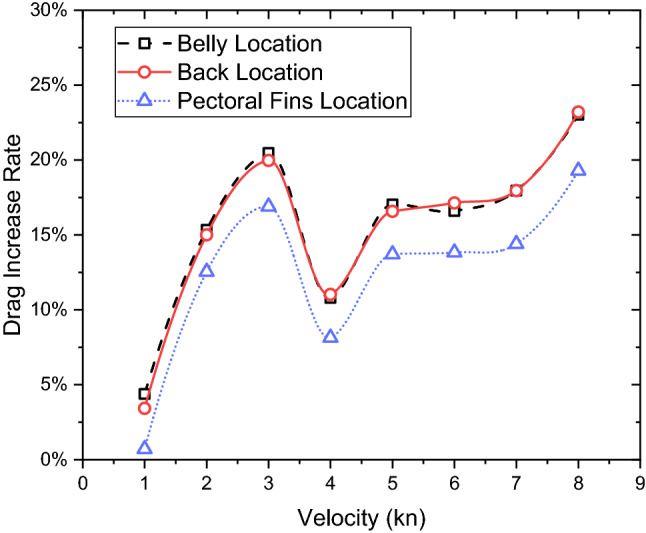
The increase rate of the total drag on the shark plus remora in different velocities.

Figure 26 shows the total drag increase rate of the shark and the remora together for the different attachment locations. These three locations have a similar trend from 1 to 8 kn velocity particularly for belly attachment and back attachment. However, attaching to the pectoral fins shows the lowest total drag increase rate.

## Conclusions

In this paper, an investigation has been conducted to study the hydrodynamic characteristics of the remora fish in attached swimming conditions. This research has primarily focused on the investigations of the effect of the boundary layer flow and the attachment locations of the remora to the body of a shark. By using computational fluid dynamics tools, the following detailed conclusions can be drawn:

1. Regarding the effect of boundary layer flow on the remora fish, a systematic study with parametrically developed boundary layer flow was conducted and found that the drag reduction rate is directly related to the boundary layer thickness. Up to 42% of drag reduction rate can be achieved at the lowest velocity ($V$=1kn) with the thickest boundary layer, therefore explaining the remora fish tends to seek a developed boundary layer when attaching to the body shark.
2. In terms of the attachment locations, the most frequent locations are: the belly, the back and the pectoral fin of the shark. It is found that the belly and the back locations are the regions with the lowest velocities and adverse pressure gradient regions. Therefore, not only the boundary layer, but also the flow characteristics in the attachment locations are determining factors in the drag reduction rate of the remora.
3. The drag reductions of the belly attachment case and the back attachment case reached 61% and 59% respectively. The drag reduction rate increases with the speed for these two locations, whereas the pectoral fin location showed the opposite trend, dropping and converging to a 29% drag reduction rate.
4. When remora attaches to the shark, it can be noted that the total drag increases and the increase rate rises with the speed. At the highest tested velocity (8 kn) and at the belly and the back, the drag increase rate reaches 23%. Contrarily, when the remora is attached to the pectoral fin, the increase of the total drag is 18%.

With the achieved comprehensive understanding of the remora’s swimming behaviour, a design study will be conducted to hydrodynamically design a remora fish inspired AUV. The above results presented here will form the foundations to investigate the feasibility of dynamically docking a novel remora-inspired AUV to a submerged mother vehicle.
